# Alcohol Consumption Patterns and Peripheral Arterial Stiffness in Young Adults: Sex-Specific Findings from the EVA-Adic Study

**DOI:** 10.3390/nu18030411

**Published:** 2026-01-26

**Authors:** Alberto Vicente-Prieto, Cristina Lugones-Sánchez, Sara Vicente-Gabriel, Cristina Saldaña-Ruiz, Susana González-Sánchez, Sandra Conde-Martín, Emiliano Rodriguez-Sanchez, Luis García-Ortiz, Marta Gómez-Sánchez, Leticia Gómez-Sánchez, Manuel Angel Gómez-Marcos, EVA-Adic Investigators Group

**Affiliations:** 1 Primary Care Research Unit of Salamanca (APISAL), Salamanca Primary Care Management, Institute of Biomedical Research of Salamanca (IBSAL), 37005 Salamanca, Spain; cristinals@usal.es (C.L.-S.); u127891@usal.es (S.V.-G.); csaldanar@saludcastillayleon.es (C.S.-R.); gongar04@gmail.com (S.G.-S.); sandraconde@usal.es (S.C.-M.); emiliano@usal.es (E.R.-S.); lgarciao@usal.es (L.G.-O.); martagmzsnchz@gmail.com (M.G.-S.); leticiagmzsnchz@gmail.com (L.G.-S.); magomez@usal.es (M.A.G.-M.); 2 Salamanca Primary Care Management, Castilla and León Health Service-SACYL, 37005 Salamanca, Spain; 3 Research Network on Chronicity, Primary Care and Health Promotion (RICAPPS), 37005 Salamanca, Spain; 4 Intensive Care Unit, Salamanca University Hospital, P. of San Vicente, 37007 Salamanca, Spain; 5 Department of Medicine, University of Salamanca, 28046 Salamanca, Spain; 6 Home Hospitalization Service, Marqués of Valdecilla University Hospital, s/n, 39008 Santander, Spain; 7 Emergency Service, University Hospital of La Paz, P. of Castellana, 261, 28046 Madrid, Spain

**Keywords:** alcohol consumption, arterial stiffness, young adults, cardiovascular risk, alcoholic beverages, vascular function

## Abstract

***Background***: The relationship between alcohol consumption and vascular function remains controversial. This study aimed to examine the association between total alcohol intake, type of alcoholic beverage, and arterial stiffness across different vascular territories in young Spanish adults, with special attention to sex-specific patterns. ***Methods****:* A descriptive cross-sectional study was conducted. Using consecutive non-probability sampling, 501 participants (222 men and 279 women) aged 18–34 years, were recruited from the urban population of Salamanca. Alcohol consumption was assessed using a standardized questionnaire and quantified in grams per week overall and by different types of drinks (wine, beer or spirits drinks). Arterial stiffness was evaluated using pulse pressure (PP), carotid–femoral pulse wave velocity (cf-PWV), brachial–ankle pulse wave velocity (ba-PWV), cardio–ankle vascular index (CAVI), and central augmentation index corrected to a heart rate of 75 beats per minute (CAIx75). ***Results***: The mean age of the sample was 26.58 ± 4.40 years, and was significantly higher in men than in women (27.04 ± 4.41 vs. 26.22 ± 4.37 years; *p* = 0.040). The mean values for vascular function parameters were as follows: PP 42.86 ± 8.45 mmHg, cf-PWV 5.60 ± 1.29 m/s, ba-PWV 38 10.80 ± 1.01 m/s, CAVI 6.13 ± 0.75, and CAIx75 7.71 ± 19.74. Participants reporting alcohol consumption showed lower ba-PWV values compared with abstainers, while no consistent associations were observed for central arterial stiffness parameters. In sex-stratified analyses, higher total alcohol intake (β = −0.002, 95% CI: −0.004–−0.001), as well as beer (β = −0.004, 95% CI: −0.007–−0.001), and spirit consumption (β = −0.004, 95% CI: −0.006–−0.001), were inversely associated with ba-PWV exclusively in men. In women, spirit consumption was positively associated with CAIx75 (β = 0.044, 95% CI: 0.006–0.081). The magnitude of the observed differences in ba-PWV was modest and occurred in a predominantly low-risk population. ***Conclusions***: In young adults, alcohol consumption was associated with differences in peripheral arterial stiffness, primarily reflected by ba-PWV, with clear sex-specific patterns. These findings do not support a causal or protective effect of alcohol consumption and should be interpreted cautiously due to the cross-sectional design of the study. The results highlight the importance of considering sex and vascular territory when evaluating early markers of vascular aging in young populations. Clinical trial registration: [ClinicalTrials.gov], identifier [NCT05819840].

## 1. Introduction

Alcohol is a psychoactive substance widely consumed worldwide, which is why its effects have been extensively researched. Alcohol consumption between 2003 and 2018 showed a steady upward trend globally [1]. The prevalence of alcohol consumption among young adults aged 18 to 34 in Spain is 90.3% [2], with high prevalence rates also observed in the European Union [3] and worldwide [4]. One explanation for these data is the social norms that allow alcohol use for socializing and drunkenness as part of these social interactions [4].

Alcohol consumption is associated with increased arterial stiffness [5,6,7,8], and increased blood pressure [9,10,11,12,13]. It also increases cardiovascular risk (CVR) and mortality [6,9,12,14,15]. Some studies have shown that the relationship between the incidence of cardiovascular disease and arterial stiffness describes a J-curve with respect to the amount of alcohol intake [14,16,17,18]. However, this interpretation has been increasingly questioned. Recent reviews, scientific statements, and clinical guidelines emphasize that previously reported cardiovascular benefits of low-to-moderate alcohol consumption are largely explained by residual confounding, selection bias, and healthier lifestyle patterns among moderate drinkers, rather than a true biological protective effect [9,13,19]. Moreover, current hypertension guidelines highlight the adverse effects of alcohol on blood pressure and vascular health even at relatively low levels of consumption, reinforcing the absence of a cardioprotective threshold [20,21].

Furthermore, few studies have analyzed the relationship between the type of alcohol and arterial stiffness, not to mention that some studies have attributed the benefits of low to moderate consumption to the polyphenols found in red wine [22]. Nevertheless, alcoholic beverages differ not only in their ethanol content but also in their non-alcoholic components and associated drinking patterns. Although wine—particularly red wine—has traditionally received greater attention due to its polyphenol content, beer contains distinct bioactive compounds derived from hops and barley. Conversely, spirits are characterized by higher ethanol concentrations and are more frequently associated with episodic or binge drinking patterns, which may exert unfavorable metabolic and vascular effects [9,12,23]. Despite these differences, evidence comparing beverage-specific associations with arterial stiffness remains limited and inconsistent, and is largely restricted to older populations and central arterial stiffness measures [6,16,17,18].

Increased arterial stiffness is a physiological process that occurs with age. An accelerated increase in arterial stiffness is considered a marker of vascular aging [24]. Central arterial stiffness is measured non-invasively using carotid–femoral pulse wave velocity (cf-PWV), peripheral stiffness is measured using arm-ankle pulse wave velocity (ba-PWV), central and peripheral measured by the cardio–ankle vascular index (CAVI), and systemic stiffness using the central augmentation index corrected for a heart rate of 75 beats per minute (CAIx75); together, these parameters provide a comprehensive assessment of the vascular tree and are independent predictors of cardiovascular disease and mortality [25,26,27].

Although arterial stiffness is more clinically relevant in older adults, vascular stiffening is a progressive process that begins early in life. Early alterations in arterial stiffness during young adulthood may reflect the initial impact of modifiable lifestyle-related exposures—such as alcohol consumption—on the vascular system, preceding the development of overt cardiovascular disease [24,28]. Therefore, the study of arterial stiffness in young adults may help identify early markers of accelerated vascular aging at a stage when preventive strategies could be particularly effective.

Collectively, these considerations highlight a significant gap in the literature regarding the association between alcohol consumption patterns—including beverage type—and arterial stiffness across different vascular territories in young adults, as well as potential sex-specific differences in these associations.

Therefore, the objective of the present study was to examine the association between total alcohol consumption and specific types of alcoholic beverages with arterial stiffness across the vascular tree in young Spanish adults. We hypothesized that alcohol consumption would be associated with arterial stiffness parameters in a sex-specific manner, with differential associations observed according to beverage type, and that these associations would be more evident for peripheral arterial stiffness than for central measures.

## 2. Materials and Methods

### 2.1. Design

The results of this study are part of the EVA-Adic study, the protocol for which was previously published [29]. The EVA-Adic study is a descriptive cross-sectional observational study conducted at the Primary Care Research Unit of Salamanca (APISAL). It is registered at ClinicalTrials.gov under registration number NCT05819840 (Registration date: 29 July 2023).

### 2.2. Study Population

Through consecutive sampling, 501 people from the urban health area of Salamanca were recruited. The inclusion criteria were: being between 18 and 34 years of age and signing the informed consent form to participate in the study. The exclusion criteria were: being in a terminal situation and being unable to travel to the research unit to undergo the tests. Participants were not excluded on the basis of a previous diagnosis of cardiovascular disease.

### 2.3. Sample Size Calculation

Using the GRANMO software version 7.2 and considering ba-PWV as the primary outcome: Accepting an alpha risk of 0.05 and a statistical power greater than 0.80 in a two-sided test, 134 subjects are required in Group 1 (non-drinkers) and 268 in Group 2 (drinkers) to detect a difference equal to or greater than 0.3 units. A common standard deviation for ba-PWV of 1.01 is assumed. An anticipated loss to follow-up rate of 0% has been estimated.

### 2.4. Variables and Measurement Instruments

All measurements and tests were performed on each participant within a period of less than 8 days. All tests were performed by 3 previously trained researchers in a standardized manner, with quality being assessed by an independent researcher. All vascular assessments were performed under standardized environmental conditions, during a single evaluation period for each participant, and after a minimum 12 h abstinence from alcohol, tobacco, and caffeine. Measurements were performed sequentially.

#### 2.4.1. Sociodemographic Variables and Personal History

Age and sex were recorded at the time of inclusion in the study using a structured clinical interview.

#### 2.4.2. Alcohol Consumption

Alcohol intake was assessed using a structured, self-administered questionnaire that recorded the quantity and type of alcohol usually consumed during a week. The amount of alcohol consumed was measured in grams/week. Alcohol use was assessed using the Alcohol Use Disorders Identification Test (AUDIT), which identifies the type of consumption: low risk (0–7 points), medium risk (8–15 points), high risk (16–19 points), dependence (20 or more points [30]. Alcohol consumption: those who consume alcohol more than once a week. This group was then subdivided according to the type of alcohol consumed: beer, wine, and spirits.

#### 2.4.3. Adherence to the Mediterranean Diet

The Mediterranean diet was assessed using the Mediterranean Diet Adherence Screener (MEDAS), which has been validated for the Spanish population [31].

#### 2.4.4. Tobacco Consumption

Smoking habits were studied using the standardized four-question questionnaire from the WHO MONICA study [32]. In addition, the number of years they had been smoking and whether they were ex-smokers were recorded.

#### 2.4.5. Physical Activity

Physical activity was calculated using the International Physical Activity Questionnaire-Short Form (IPAQ-SF) [33]. This consists of seven questions about the type of physical activity performed during the last seven days and the total time spent on these activities. The result is the metabolic equivalent of the task per minute per week (METs-min/week).

#### 2.4.6. Arterial Stiffness

Arterial stiffness was studied by measuring carotid–femoral pulse wave velocity (cf-PWV), arm-to-ankle pulse wave velocity (ba-PWV), cardio–ankle vascular index (CAVI), and central augmentation index corrected for a heart rate of 75 beats per minute (CAIx75).

We used the SphygmoCor device (AtCor Medical Pty Ltd., head office, Sydney, Australia) to measure cf-PWV and CAIx75. The measurement was performed with the patient in the supine position. The pulse wave at the carotid and femoral levels was calculated by estimating the delay between the R wave of the ECG and calculating the cf-PWV. The distance was calculated with a tape measure from the sternal notch to the sensor at the carotid and femoral levels [34].

With a sensor on the radial artery to monitor the pulse wave, the following were obtained: the pressure at the inflection point of the pulse wave (P1) and the pressure at the maximum peak of the wave, maximum pressure during systole (P2). The difference between these two pressures (P2 − P1) is called the augmentation pressure (AP). We obtained the CAIx by dividing AP/PP. To standardize the measurement and compare the results, we calculated the CAIx75 using the following equation: CAIx75 = CAIx − қ x (HR − 75) [35], where HR refers to the patient’s heart rate.

Ba-PWV and CAVI were measured with the VaSera VS-2000 device (Fukuda Denshi Co., Ltd., Tokyo, Japan). Electrodes were placed on the arms and legs. Measurements were taken with the patient silent and still. A sensor was placed to detect heart sounds in the second intercostal space. To calculate CAVI, we used the following equation: stiffness parameter β = 2ρ × 1/(SBP − DBP) × ln(SBP/DBP) × PWV, where ρ is blood density and PWV is measured between the aortic valve and the ankle. The measurements were considered correct after three heartbeats [36]. Ba-PWV was calculated using the following equation: ba-PWV = (0.5934 × height (cm) + 14.4724)/tba, where tba is the time interval between the ankle and arm waves [37]. CAVI values were divided into: normal (CAVI < 8), normal-high (8 ≤ CAVI > 9), abnormal (CAVI ≥ 9) [36].

#### 2.4.7. Cardiovascular Risk Factors

*Analytical variables:* Venous blood samples were taken between 8 a.m. and 9 a.m., with the patient fasting and having consumed no alcohol, tobacco, or caffeine in the previous 12 h. The samples were collected at APISAL. These samples were analyzed for glucose, cholesterol, HDL cholesterol, LDH cholesterol, triglycerides, glucose and uric acid. The samples were coded and analyzed using standardized laboratory techniques.

*Blood pressure measurement*: Blood pressure was measured three times in a row using a validated sphygmomanometer, model Omron M10-IT (Omron Healthcare, Kyoto, Japan). Measurements were taken on the patient’s dominant arm while seated after at least 5 min of rest and using a cuff of the appropriate size. The recommendations of the European Society of Hypertension (ESH) [20] were followed. In this way, we obtained systolic (SBP) and diastolic (DBP) blood pressure. Clinical pulse pressure (CPP) was obtained by subtracting systolic pressure from diastolic pressure [38].

*Anthropometric variables:* Height was measured in centimeters with a calibrated tape measure (Seca 222, Medical Scale and Measurement Systems, Birmingham, UK), with the patient standing barefoot with their back to the wall and breathing in. Waist circumference was measured with a flexible tape measure, keeping it parallel to the floor above the iliac crests, at the end of exhalation, with the patient standing upright and without clothing. These measurements were taken following the recommendations of the Spanish Society for the Study of Obesity (SEEDO) [39]. Weight was measured in kilograms using the InBody 230^®^ (InBody Co., Ltd., Seoul, Republic of Korea) instrument, following the manufacturer’s instructions. Body mass index (BMI) was calculated by dividing the patient’s weight by the square of their height in centimeters.

### 2.5. Statistical Analysis

Continuous variables are shown as mean ± SD, and Student’s *t*-test was used to estimate the differences between them. Categorical variables are shown as number and percentage, and the Chi-square test was used to estimate the differences between them. Multiple regression analysis was used to examine the relationship between arterial stiffness measurements and different behavioral addictions. In the multiple regression analysis, we used alcohol consumption in grams per week, beer consumption in grams per week, wine consumption in grams per week, and distilled beverage consumption in grams per week as independent variables. As dependent variables, we used clinical PP in mmHg, cf-PWV and ba-PWV scores in meters/second (m/s), CAIx75, and CAVI. As adjustment variables, we used age in years, coded sex (sex: male = 0; female = 1), lifestyle: diet with the average score on the MEDAS questionnaire; physical activity was calculated using the International Physical Activity Questionnaire-Short Form (IPAQ-SF), measuring the total time spent on such activities. We obtained the metabolic equivalent of the task per minute per week (METs-min/week) and sleep quality and quantity using the total score of the Pi test, waist circumference measured in cm, blood glucose, uric acid, triglycerides, and LDL cholesterol in mg/dL. Covariates included in the multivariable models were selected a priori based on clinical and epidemiological evidence and biological plausibility regarding their association with arterial stiffness and vascular function, rather than on data-driven statistical selection procedures. These covariates represent established cardiovascular risk factors and lifestyle-related variables known to influence arterial stiffness. Handling of extreme values and multicollinearity: No a priori exclusion of extreme values was applied for alcohol intake expressed in grams per week, as these values reflect real consumption patterns in the study population. Alcohol consumption variables were analyzed as continuous measures. To avoid multicollinearity, total alcohol intake and beverage-specific consumption variables were evaluated in separate regression models and were not entered simultaneously as predictors. The statistical program used was SPSS for Windows version 28.0. (IBM Corp, Armonk, NY, USA). We considered values of *p* < 0.05 to be significant. The staff responsible for performing the statistics were blinded to the patient’s clinical data. All statistical analyses were performed globally and by gender.

### 2.6. Ethical Principles

The Salamanca Health Area Drug Research Ethics Committee approved this project on 10 July 2021 (Reference Code CEIm Ref. PI 2021 088671048) and on 24 July 2023 (Reference Code CEIm Ref. PI 2023 071332). The recommendations of the Declaration of Helsinki [40] and the WHO were followed during the conduct of the study. Confidentiality was ensured in accordance with Organic Law 3/2018 of 5 December on the Protection of Personal Data and Guarantee of Digital Rights and Regulation (EU) 2016/679 of the European Parliament and of the Council of 27 April 2016 (GDPR). All participants signed the informed consent form before being included in the study and after being informed about all the tests and questionnaires that would be administered to them.

## 3. Results

### 3.1. Characteristics of Participants

The main characteristics of the study participants are shown overall and broken down by gender in Table 1. More women than men were included (279 vs. 222, *p* < 0.05). Men consume more alcohol and cigarettes and engage in more physical activity than women. Women have a higher Mediterranean diet score than men. Men have higher levels of LDL cholesterol, HDL cholesterol, triglycerides, blood pressure, and obesity parameters than women.

### 3.2. Overall and Alcohol-Type–Specific Consumption

**Figure 1:**
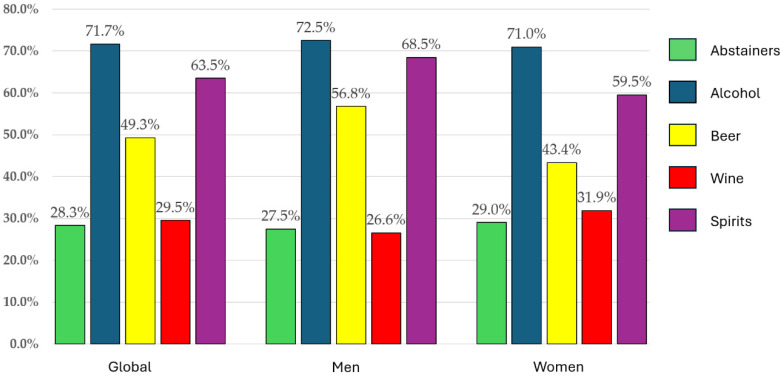
Overall and alcohol-type–specific consumption patterns, expressed as percentages, in the total sample and stratified by sex. Percentage distribution of overall alcohol consumption and specific alcohol types in the total sample and by sex.

Overall and alcohol-type–specific consumption patterns are presented in Figure 1. No differences were observed in total alcohol intake between men and women (*p* = 0.765). Regarding the most consumed beverage type, spirits were the most consumed across the entire sample. Consumption of both spirits and beer was higher in men than in women (*p* = 0.040 and *p* = 0.003, respectively). No differences were found in wine consumption between the sexes.

### 3.3. Arterial Stiffness

Arterial stiffness measurements according to overall alcohol consumption and alcohol type are presented in Table 2. A significant difference was observed in ba-PWV arterial stiffness between the two groups (*p* = 0.035).

Table 3 presents the analysis of arterial stiffness stratified by the type of alcohol consumed. Significant differences were observed in PP and ba-PWV among beer consumers. Post hoc tests revealed that differences in PP lay between beer consumers and consumers of other alcoholic beverages. Regarding ba-PWV, differences were found between non-consumers and consumers of other alcoholic beverages. Post hoc analyses did not reach statistical significance after adjustment for multiplicity among the different groups regarding spirit consumption. No differences in arterial stiffness were observed regarding wine consumption.

Exploratory correlation analyses between alcohol consumption variables and arterial stiffness parameters are provided in the Supplementary Material (Figures S2–S4). These analyses were descriptive in nature and did not modify the interpretation of the multivariable regression results presented above.

Table 4 displays the linear regression analysis considering alcohol consumption as the independent variable and vascular function parameters as dependent variables. The model was adjusted for the following confounding factors, which were selected based on previous evidence: fasting glucose levels, LDL cholesterol, triglycerides, uric acid, age, waist circumference, total METs-min/week, adherence to the Mediterranean diet, and smoking status. In the overall sample, alcohol consumption was not associated with arterial stiffness. In men, higher alcohol consumption was associated with lower ba-PWV values (*p* < 0.001). Conversely, in women, higher alcohol consumption showed no association with any vascular function parameter, although CAIx75 approached statistical significance (*p* = 0.050).

In the analysis stratified by type of alcoholic beverage, beer and spirit consumption were associated with lower ba-PWV values in men (*p* = 0.031 and *p* = 0.005, respectively). Conversely, in women, spirit consumption was associated with higher CAIx75 values (*p* = 0.022). No significant associations were found between wine consumption and vascular parameters in any of the subgroups.

### 3.4. At-Risk Drinkers Based on the AUDIT

**Figure 2:**
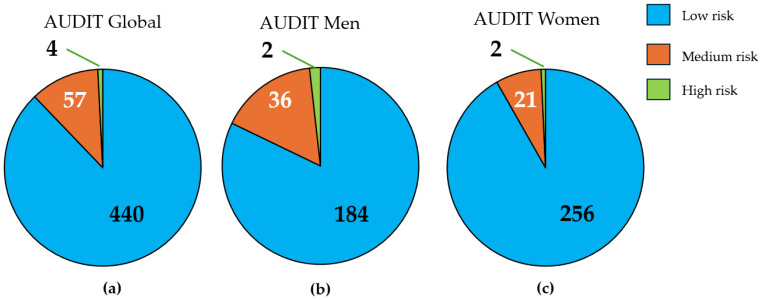
Results of the AUDIT. The graphs display the total population values according to alcohol consumption risk in the overall sample (**a**), men (**b**), and women (**c**).

Figure 2 displays the classification of the study population based on the results of the AUDIT. We observed that the majority of the study population presented a low risk according to the AUDIT (87.82%; 82.88% in men and 97.75% in women). High risk was identified in 0.80% of the sample (0.90% in men and 0.70% in women).

## 4. Discussion

The present study investigated the association between alcohol consumption patterns and arterial stiffness across various vascular territories in a cohort of young adults. The principal finding of the study is the association between alcohol consumption and lower peripheral arterial stiffness, as assessed by brachial–ankle pulse wave velocity (ba-PWV), in the overall sample and specifically among men. In contrast, no consistent associations were observed for parameters of central arterial stiffness. This observation constitutes the study’s primary contribution and must be interpreted within the context of a young, predominantly low-risk population.

Following adjustment for relevant demographic, anthropometric, metabolic, and lifestyle-related covariates, alcohol consumers exhibited lower ba-PWV values compared to abstainers, particularly among men. Conversely, no consistent associations were found for cf-PWV or CAVI. These results highlight the importance of prioritizing peripheral arterial stiffness as a potentially more sensitive vascular marker in this specific population, while avoiding the overinterpretation of secondary or inconsistent findings across other vascular indices.

Crucially, although the associations observed for ba-PWV were statistically significant, their magnitude was modest. Given the young age of the participants and their generally low cardiovascular risk profile, the clinical and prognostic relevance of these differences remains uncertain. Consequently, the observed variations in ba-PWV should be interpreted as reflecting variability in early vascular function rather than serving as evidence of clinically meaningful vascular protection or reduced cardiovascular risk.

When alcohol consumption was analyzed by beverage type, inverse associations with ba-PWV were identified for beer and spirits, whereas wine consumption exhibited weaker and less consistent associations. Although wine—particularly red wine—has traditionally received considerable attention due to its polyphenol content and potential cardiovascular benefits [22], the present study did not distinguish between red and white wine consumption. This limitation may partly explain the lack of consistent associations regarding wine intake. Furthermore, alcoholic beverages differ not only in their non-alcoholic bioactive components but also in ethanol concentration and associated drinking patterns, which may influence vascular outcomes through multiple and potentially opposing mechanisms.

Several biological mechanisms have been proposed to explain the relationship between alcohol consumption and vascular function, including the modulation of endothelial nitric oxide bioavailability, oxidative stress, inflammation, and lipid metabolism [9,12,22]. However, as these mechanisms cannot be directly evaluated or confirmed within the context of the present cross-sectional study, they must be regarded as speculative. Accordingly, these explanations should be viewed as plausible hypotheses derived from prior experimental or longitudinal research, rather than as mechanisms inferred directly from the current findings.

The discrepancy between the associations observed for peripheral arterial stiffness (ba-PWV) and the null findings for central arterial stiffness parameters warrants careful consideration. Peripheral arterial stiffness measures may be more sensitive to early functional changes in muscular arteries, particularly in young adults, whereas central arterial stiffness more closely reflects structural alterations in elastic arteries that typically develop later in life [24,25,26,27,28]. Factors such as differences in measurement sensitivity, age-related vascular physiology, and statistical power may also contribute to the divergence of findings across vascular territories.

Several limitations of the present study must be acknowledged. First, the cross-sectional design precludes the determination of causality and prevents conclusions regarding potential protective or harmful effects of alcohol consumption. Residual confounding cannot be entirely excluded, particularly for lifestyle-related exposures such as alcohol intake, which are influenced by complex behavioral, social, and cultural factors. Second, alcohol consumption was self-reported, which may introduce misclassification bias. Third, the study did not distinguish between regular and episodic (binge) drinking patterns, nor between specific types of wine, which may have influenced the observed associations. Finally, although the sample size was adequate to detect small-to-moderate differences in peripheral arterial stiffness, the study may have been underpowered to detect subtle associations with central arterial stiffness parameters.

Despite these limitations, the study possesses several strengths, including the comprehensive assessment of arterial stiffness across multiple vascular territories, the evaluation of beverage-specific consumption patterns, and the focus on a young adult population—a group in whom early vascular changes remain relatively understudied.

In conclusion, alcohol consumption was associated with lower peripheral arterial stiffness, as assessed by ba-PWV, with clear sex-specific patterns. These associations were modest in magnitude and should not be interpreted as definitive evidence of a protective effect of alcohol consumption. The findings highlight the importance of considering both vascular territory and sex when examining early markers of vascular aging, and emphasize the need for longitudinal studies to clarify the clinical relevance and underlying mechanisms of these associations.

### Future Perspectives

Although the findings of this study are novel within the field, further research is required to determine the long-term impact of alcohol consumption—and the specific type of alcohol consumed—on the vascular health of young adults. Prospective longitudinal studies are needed to elucidate whether ba-PWV can be considered an early marker of accelerated vascular aging. Furthermore, employing novel methods to record alcohol consumption, such as validated mobile applications [41], could serve as a strategy to mitigate recall bias when patients self-report their intake over the preceding days or months. Future studies should ideally integrate arterial stiffness measurements with biomarkers of inflammation, oxidative stress, and endothelial dysfunction.

Other potential lines of research could address consumption patterns (chronic vs. binge drinking) and their impact on arterial stiffness in the young adult population. Additionally, studies specifically designed to elucidate sex-specific differences in the effect of alcohol on arterial stiffness would also be a valuable subject of investigation.

## 5. Conclusions

In conclusion, alcohol consumption was associated with differences in peripheral arterial stiffness, as assessed by ba-PWV, with clear sex-specific patterns. These associations were modest in magnitude and should not be interpreted as evidence of a protective effect of alcohol consumption. The findings highlight the importance of considering vascular territory and sex when examining early markers of vascular aging and emphasize the need for longitudinal studies to clarify the clinical relevance and underlying mechanisms of these associations.

## Figures and Tables

**Table 1:** General characteristics of the subjects analyzed in the study as a whole and divided by gender.

Variable	Global (n = 501)	Men (n = 222)	Women (n = 279)	*p*
Age (years)	26.58 ± 4.40	27.04 ± 4.41	26.22 ± 4.37	0.040
Lifestyle				
Total alcohol (g/w)	48.80 ± 73.59	61.54 ± 91.14	38.66 ± 53.90	0.001
Beer (g/w)	19.39 ± 32.82	26.31 ± 41.07	13.89 ± 22.97	<0.001
Wine (g/w)	7.74 ± 16.66	7.16 ± 18.01	7.67 ± 15.52	0.735
Spirits (g/w)	21.96 ± 47.64	28.08 ± 57.96	17.10 ± 36.84	0.015
Active smokers (n, %)	70 ± 14.0	28 ± 12.61	42 ± 15.05	0.434
MD (total score)	7.44 ± 2.01	6.96 ± 2.06	7.81 ± 1.88	<0.001
METs-minute/week	2651 ± 2490	3159 ± 3130	2248 ± 1730	<0.001
Risk factor				
Total cholesterol (mg/dL)	170.16 ± 28.66	170.98 ± 29.77	169.52 ± 27.79	0.573
LDL (mg/dL)	96.13 ± 25.97	101.83 ± 28.47	91.50 ± 22.78	<0.001
HDL (mg/dL)	58.30 ± 12.85	52.20 ± 10.84	63.12 ± 12.26	<0.001
Tryglicerides (mg/dL)	83.11 ± 44.24	95.55 ± 55.70	73.19 ± 28.80	<0.001
Glucose (mg/dL)	81.10 ± 12.43	82.35 ± 16.20	80.09 ± 8.18	0.044
Uric acid (mg/dL)	5.04 ± 1.33	6.00 ± 1.17	4.29 ± 0.89	<0.001
SBP (mmHg)	110.94 ± 11.56	118.61 ± 10.29	104.84 ± 8.46	<0.001
DBP (mmHg)	68.08 ± 7.78	70.15 ± 8.11	66.44 ± 7.11	<0.001
BMI (kg/m^2^)	24.04 ± 3.80	25.30 ± 3.65	23.03 ± 3.63	<0.001
Waist circumference (cm)	79.39 ± 11.50	85.23 ± 10.58	74.74 ± 9.99	<0.001
Arterial stiffness				
PP (mmHg)	42.86 ± 8.45	48.47 ± 7.90	38.39 ± 5.81	<0.001
cf-PWV (m/s)	5.60 ± 1.29	5.95 ± 1.18	5.33 ± 1.30	<0.001
ba-PWV (m/s)	10.80 ± 1.01	11.20 ± 1.05	10.48 ± 0.84	<0.001
CAVI	6.13 ± 0.75	6.16 ± 0.75	6.10 ± 0.74	0.341
CAIx75	7.71 ± 19.74	6.21 ± 26.27	8.90 ± 12.23	0.133

The values are the means and standard deviations of continuous data and the numbers and proportions of categorical data. MD: Mediterranean diet. MET: metabolic equivalent of task. g/w: grams per week. LDL: low density lipoprotein cholesterol. HDL: High density lipoprotein cholesterol. SBP: systolic blood pressure. DBP: diastolic blood pressure. PP: clinical pulse pressure. BMI: body mass index. CAVI: cardio–ankle vascular index. cf-PWV: carotid–femoral pulse wave velocity. ba-PWV: brachial–ankle pulse wave velocity. CAIx75: central augmentation index corrected for a heart rate of 75 beats per minute. *p* value: differences between men and women.

**Table 2:** Arterial stiffness measurements in subjects with and without alcohol consumption.

Variable	Consumers (n = 359)	Abstainers (n = 142)	*p* Value
PP (mmHg)	42.97 ± 8.18	42.58 ± 9.12	0.645
cf-PWV (m/s)	5.60 ± 1.32	5.63 ± 1.19	0.806
ba-PWV (m/s)	10.74 ± 0.97	10.95 ± 1.07	0.035
CAVI	6.15 ± 0.70	6.08 ± 0.85	0.384
CAIx75	6.75 ± 12.51	10.16 ± 31.36	0.084

Values are expressed as mean standard deviation (SD) of arterial stiffness measurements stratified by alcohol consumption. PP: clinical pulse pressure; cf-PWV: carotid–femoral pulse wave velocity; ba-PWV: brachial–ankle pulse wave velocity; CAVI: cardio–ankle vascular index; CAIx75: central augmentation index adjusted for a heart rate of 75 bpm. m/s: meters per second. *p*-value: differences between alcohol consumers and non-consumers.

**Table 3:** Arterial stiffness measurements in subjects with and without alcohol consumption stratified by the type of alcoholic beverage.

**Beer**	**Consumers** **(n = 247)**	**Abstainers** **(n = 142)**	**Other Beverages** **(n = 112)**	***p* Value**
PP (mmHg)	43.88 ± 8.28	42.58 ± 9.12	40.95 ± 7.60	0.008
cf-PWV (m/s)	5.66 ± 1.50	5.63 ± 1.19	5.46 ± 0.77	0.395
ba-PWV (m/s)	10.79 ± 0.96	10.95 ± 1.07	10.63 ± 0.99	0.043
CAVI	6.13 ± 0.73	6.08 ± 0.85	6.19 ± 0.63	0.488
CAIx75	6.42 ± 12.17	10.16 ± 31.36	7.49 ± 13.27	0.201
**Wine**	**Consumers** **(n = 148)**	**Abstainers** **(n = 142)**	**Other Beverages** **(n = 41)**	***p*** **Value**
PP (mmHg)	42.71 ± 7.60	42.58 ± 9.12	43.15 ± 8.58	0.797
cf-PWV (m/s)	5.48 ± 1.60	5.63 ± 1.19	5.68 ± 1.09	0.345
ba-PWV (m/s)	10.73 ± 0.93	10.95 ± 1.07	10.75 ± 1.01	0.106
CAVI	6.12 ± 0.69	6.08 ± 0.85	6.17 ± 0.71	0.529
CAIx75	7.37 ± 13.20	10.16 ± 31.36	6.32 ± 12.02	0.199
**Spirits**	**Consumers** **(n = 148)**	**Abstainers** **(n = 142)**	**Other Beverages** **(n = 41)**	***p*** **Value**
PP (mmHg)	43.33 ± 8.18	42.58 ± 9.12	40.20 ± 7.71	0.074
cf-PWV (m/s)	5.64 ± 1.38	5.63 ± 1.19	5.25 ± 0.64	0.210
ba-PWV (m/s)	10.76 ± 0.98	10.95 ± 1.07	10.55 ± 0.88	0.046
CAVI	6.15 ± 0.71	6.08 ± 0.85	6.13 ± 0.67	0.633
CAIx75	6.60 ± 11.92	10.16 ± 31.36	7.93 ± 16.59	0.208

Values are expressed as mean standard deviation (SD) of arterial stiffness measurements stratified by the type of alcoholic beverage consumed. PP: clinical pulse pressure; cf-PWV: carotid–femoral pulse wave velocity; ba-PWV: brachial–ankle pulse wave velocity; CAVI: cardio–ankle vascular index; CAIx75: central augmentation index adjusted for a heart rate of 75 bpm. m/s: meters per second. *p*-value: differences among consumers, non-consumers, and consumers of other alcoholic beverages.

**Table 4:** Association analysis between alcohol intake and vascular function parameters in the overall sample and stratified by sex.

	Global				Men				Women			
Parameters												
Alcohol	β	CI	95%	*p* value	β	CI	95%	*p* value	β	CI	95%	*p* value
PP (mmHg)	0.006	−0.004	0.015	0.219	0.002	−0.010	0.013	0.804	0.006	−0.007	0.018	0.389
cf-PWV (m/s)	0.001	−0.001	0.001	0.933	−0.001	−0.003	0.001	0.368	0.001	−0.001	0.003	0.162
ba-PWV (m/s)	−0.001	−0.002	0.000	0.113	−0.002	−0.004	−0.001	0.003	0.001	−0.001	0.003	0.338
CAVI	0.001	−0.009	0.009	0.983	−0.007	−0.018	0.004	0.226	0.009	−0.006	0.024	0.226
CAIx75	−0.015	−0.040	0.010	0.240	−0.030	−0.071	0.011	0.145	0.024	−0.002	0.050	0.067
Beer	β	CI	95%	*p* value	β	CI	95%	*p* value	β	CI	95%	*p* value
PP (mmHg)	0.010	−0.011	0.031	0.335	0.008	−0.018	0.034	0.537	0.006	−0.025	0.036	0.712
cf-PWV (m/s)	−0.001	−0.004	0.002	0.469	−0.003	−0.006	0.001	0.197	0.003	−0.002	0.007	0.297
ba-PWV (m/s)	−0.001	−0.004	0.001	0.367	−0.004	−0.007	−0.001	0.031	0.003	−0.001	0.008	0.120
CAVI	−0.001	−0.003	0.001	0.570	−0.002	−0.004	0.001	0.179	0.001	−0.002	0.005	0.419
CAIx75	−0.046	−0.102	0.010	0.110	−0.063	−0.151	0.026	0.167	−0.005	−0.068	0.057	0.864
Wine	β	CI	95%	*p* value	β	CI	95%	*p* value	β	CI	95%	*p* value
PP (mmHg)	0.032	−0.009	0.072	0.123	0.010	−0.047	0.068	0.724	0.040	−0.005	0.085	0.085
cf-PWV (m/s)	0.002	−0.003	0.008	0.447	0.001	−0.008	0.009	0.845	0.003	−0.005	0.010	0.499
ba-PWV (m/s)	0.000	−0.006	0.005	0.876	−0.004	−0.012	0.004	0.306	0.003	−0.004	0.009	0.421
CAVI	0.001	−0.003	0.005	0.599	−0.001	−0.007	0.004	0.627	0.002	−0.003	0.007	0.430
CAIx75	−0.024	−0.131	0.082	0.653	−0.065	−0.262	0.133	0.519	0.054	−0.038	0.146	0.252
Spirits	β	CI	95%	*p* value	β	CI	95%	*p* value	β	CI	95%	*p* value
PP (mmHg)	0.005	−0.009	0.020	0.495	−0.002	−0.021	0.017	0.864	0.003	−0.016	0.022	0.750
cf-PWV (m/s)	0.000	−0.002	0.002	0.720	−0.001	−0.004	0.002	0.570	0.002	−0.001	0.005	0.256
ba-PWV (m/s)	−0.002	−0.004	0.000	0.077	−0.004	−0.006	−0.001	0.005	0.000	−0.003	0.003	0.909
CAVI	0.000	−0.001	0.002	0.816	−0.001	−0.003	0.001	0.422	0.001	−0.001	0.003	0.347
CAIx75	−0.011	−0.049	0.028	0.578	−0.037	−0.103	0.028	0.266	0.044	0.006	0.081	0.022

Association analysis between behavioral addiction scores and arterial stiffness measurements in the overall sample and stratified by sex. PP: clinical pulse pressure; cf-PWV: carotid–femoral pulse wave velocity; ba-PWV: brachial–ankle pulse wave velocity; CAVI: cardio–ankle vascular index; CAIx75: central augmentation index adjusted for a heart rate of 75 bpm. m/s: meters per second. *p*-value: association between the parameter and the overall group, men, or women.

## Data Availability

The data supporting the findings of this study are available on ZE-NODO under the DOI. 10.5281/zenodo.14282873.

## Supplementary Materials

The following supporting information can be downloaded at: https://www.mdpi.com/article/10.3390/nu18030411/s1, Figure S1: Flowchart of the sample selection process; Figure S2: Overall correlation between alcohol consumption and arterial stiffness; Figure S3. Overall correlation between alcohol consumption and arterial stiffness in men; Figure S4. Overall correlation between alcohol consumption and arterial stiffness in women.

## Abbreviations

The following abbreviations are used in this manuscript:

cf-PWV	carotid–femoral pulse wave velocity
ba-PWV	brachial–ankle pulse wave velocity
CAVI	cardio–ankle vascular index
CAIx75	central augmentation index adjusted for a heart rate of 75 bpm.
CVR	Cardiovascular risk
AUDIT	Alcohol Use Disorders Identification TEST
MEDAS	Mediterranean Diet Adherence Screener
IPAQ-SF	International Physical Activity Questionnaire-Short Form
METs-min/week	Metabolic equivalents of task (min/week)
SBP	Systolic blood pressure
DBP	Diastolic blood pressure
PP	Clinical pulse pressure
BMI	body mass index

## Appendix A

EVA-Adic Investigators Group: The members of the EVA-Adic Group are: Manuel A. Gómez Marcos, Luis García-Ortiz, Emiliano Rodríguez-Sánchez, Cristina Lugones-Sánchez, Olaya Tamayo-Morales, Susana González-Sánchez, Leticia Gómez-Sánchez, Sara M. Vicente-Gabriel, Alberto Vicente-Prieto, Sandra Conde-Martín, Marta Gómez-Sánchez, Elena Navarro Matias, Carmen Patino-Alonso, José A. Maderuelo-Fernández, Angela de Cabo-Laso, Benigna Sanchez-Salgado, and Laura Fernandez-Matas.

## References

[1] Ngo A.P., Wang X., Slater S., Chriqui J.F., Chaloupka J., Yang L., Smith L., Li Q., Shang C. (2021). Alcohol excise taxes as a percentage of retail alcohol prices in 26 oecd countries. Drug Alcohol. Depend..

[2] Observatorio Español de las Drogas y las Adicciones (2025). Informe 2025: Alcohol, Tabaco y Drogas Ilegales en España.

[3] European Union Drugs Agency (2025). Prevalence of Drug Use, Alcohol, Lifetime Prevalence, Young Adults (15–34). Statistical Bulletin 2025. https://www.euda.europa.eu/data/stats2025/gps_en.

[4] World Health Organization (2024). Global Status Report on Alcohol and Health and Treatment of Substance Use Disorders.

[5] Cypiene A., Gimzauskaite S., Rinkuniene E., Jasiunas E., Laucevicius A., Ryliskyte L., Badariene J. (2023). Effect of alcohol consumption habits on early arterial aging in subjects with metabolic syndrome and elevated serum uric acid. Nutrients.

[6] O’Neill D., Britton A., Brunner E.J., Bell S. (2017). Twenty-five-year alcohol consumption trajectories and their association with arterial aging: A prospective cohort study. J. Am. Heart Assoc..

[7] Schutte R., Zhang J., Kiran M., Ball G. (2024). Alcohol and arterial stiffness in middle-aged and older adults: Cross-sectional evidence from the uk biobank study. Alcohol. Clin. Exp. Res..

[8] Thivierge G.S., Greenlund I.M., Bigalke J.A., Smoot C.A., Carter J.R., Durocher J.J. (2025). Cardiovascular and aortic wave reflection responses to evening binge alcohol consumption. Am. J. Physiol. Heart Circ. Physiol..

[9] Piano M.R. (2017). Alcohol’s effects on the cardiovascular system. Alcohol. Res..

[10] Yu A., Cooke A.B., Scheffler P., Doonan R.J., Daskalopoulou S.S. (2021). Alcohol exerts a shifted u-shaped effect on central blood pressure in young adults. J. Gen. Intern. Med..

[11] Tasnim S., Tang C., Musini V.M., Wright J.M. (2020). Effect of alcohol on blood pressure. Cochrane Database Syst. Rev..

[12] Georgescu O.S., Martin L., Tartea G.C., Rotaru-Zavaleanu A.D., Dinescu S.N., Vasile R.C., Gresita A., Gheorman V., Aldea M., Dinescu V.C. (2024). Alcohol consumption and cardiovascular disease: A narrative review of evolving perspectives and long-term implications. Life.

[13] Piano M.R., Marcus G.M., Aycock D.M., Buckman J., Hwang C.L., Larsson S.C., Mukamal K.J., Roerecke M. (2025). Alcohol use and cardiovascular disease: A scientific statement from the american heart association. Circulation.

[14] Goel S., Sharma A., Garg A. (2018). Effect of alcohol consumption on cardiovascular health. Curr. Cardiol. Rep..

[15] Zhao J., Stockwell T., Naimi T., Churchill S., Clay J., Sherk A. (2023). Association between daily alcohol intake and risk of all-cause mortality: A systematic review and meta-analyses. JAMA Netw. Open.

[16] Gonzalez-Sanchez J., Garcia-Ortiz L., Rodriguez-Sanchez E., Maderuelo-Fernandez J.A., Tamayo-Morales O., Lugones-Sanchez C., Recio-Rodriguez J.I., Gomez-Marcos M.A., EVA Investigators (2020). The relationship between alcohol consumption with vascular structure and arterial stiffness in the spanish population: Eva study. Alcohol. Clin. Exp. Res..

[17] Arroyo-Romero S., Gomez-Sanchez L., Suarez-Moreno N., Navarro-Caceres A., Dominguez-Martin A., Lugones-Sanchez C., Tamayo-Morales O., Gonzalez-Sanchez S., Castro-Rivero A.B., Gomez-Sanchez M. (2025). Relationship between alcohol consumption and vascular structure and arterial stiffness in adults diagnosed with persistent covid: Bioicoper study. Nutrients.

[18] Del Giorno R., Maddalena A., Bassetti S., Gabutti L. (2022). Association between alcohol intake and arterial stiffness in healthy adults: A systematic review. Nutrients.

[19] National Institute on Alcohol Abuse and Alcoholism Alcohol’s Effects on Health, What Is a Standard Drink? 2025. https://www.niaaa.nih.gov/alcohols-effects-health/what-standard-drink.

[20] McEvoy J.W., McCarthy C.P., Bruno R.M., Brouwers S., Canavan M.D., Ceconi C., Christodorescu R.M., Daskalopoulou S.S., Ferro C.J., Gerdts E. (2024). 2024 esc guidelines for the management of elevated blood pressure and hypertension. Eur. Heart J..

[21] Ohya Y. (2026). The japanese society of hypertension guidelines for the management of elevated blood pressure and hypertension 2025 (jsh2025). Hypertens. Res..

[22] Moreira D.M., Martins L.F., Savas L.A., Cegielka R. (2025). Comparison of the effects of alcoholic and non-alcoholic red wine on flow-mediated dilation and brachial artery vasodilation. Int. J. Cardiovasc. Sci..

[23] Arora M., ElSayed A., Beger B., Naidoo P., Shilton T., Jain N., Armstrong-Walenczak K., Mwangi J., Wang Y., Eisele J.L. (2022). The impact of alcohol consumption on cardiovascular health: Myths and measures. Glob. Heart.

[24] Boutouyrie P., Chowienczyk P., Humphrey J.D., Mitchell G.F. (2021). Arterial stiffness and cardiovascular risk in hypertension. Circ. Res..

[25] Yue X., Chen L., Shi Y., Suo Y., Liao S., Cheang I., Gao R., Zhu X., Zhou Y., Yao W. (2024). Comparison of arterial stiffness indices measured by pulse wave velocity and pulse wave analysis for predicting cardiovascular and all-cause mortality in a chinese population. Hypertens. Res..

[26] Back M., Topouchian J., Labat C., Gautier S., Blacher J., Cwynar M., de la Sierra A., Pall D., Duarte K., Fantin F. (2024). Cardio-ankle vascular index for predicting cardiovascular morbimortality and determinants for its progression in the prospective advanced approach to arterial stiffness (triple-a-stiffness) study. EBioMedicine.

[27] Stone K., Fryer S., McDonnell B.J., Meyer M.L., Faulkner J., Agharazii M., Fortier C., Pugh C.J.A., Paterson C., Zieff G. (2024). Aortic-femoral stiffness gradient and cardiovascular risk in older adults. Hypertension.

[28] McEniery C.M., Yasmin, Hall I.R., Qasem A., Wilkinson I.B., Cockcroft J.R., ACCT Investigators (2005). Normal vascular aging: Differential effects on wave reflection and aortic pulse wave velocity: The anglo-cardiff collaborative trial (acct). J. Am. Coll. Cardiol..

[29] Vicente-Gabriel S., Lugones-Sanchez C., Tamayo-Morales O., Prieto A.V., Gonzalez-Sanchez S., Martin S.C., Gomez-Sanchez M., Rodriguez-Sanchez E., Garcia-Ortiz L., Gomez-Sanchez L. (2024). Relationship between addictions and obesity, physical activity and vascular aging in young adults (eva-adic study): A research protocol of a cross-sectional study. Front. Public. Health.

[30] Montero S.Á., Casado P.G., Cruz C.L.T.D.L., Fernandez F.B. (2001). Papel del test audit (alcohol use disorders identification test) para la detección de consumo excesivo de alcohol en atención primaria. Rev. de Med. Fam. Y Comunitaria.

[31] Schröder H., Fito M., Estruch R., Martínez-González M.A., Corella D., Salas-Salvado J., Lamuela-Raventós R., Ros E., Salaverría I., Fiol M. (2011). A short screener is valid for assessing mediterranean diet adherence among older spanish men and women. J. Nutr. Nutr. Epidemiol..

[32] Tunstall-Pedoe H. (1988). The world health organization monica project (monitoring trends and determinants in cardiovascular disease): A major international collaboration. J. Clin. Epidemiol..

[33] Lee P.H., Macfarlane D.J., Lam T.H., Stewart S.M. (2011). Validity of the international physical activity questionnaire short form (ipaq-sf): A systematic review. Int. J. Behav. Nutr. Phys. Act..

[34] Fortier C., Agharazii M. (2016). Arterial stiffness gradient. Pulse.

[35] Gatzka C.D., Cameron J.D., Dart A.M., Berry K.L., Kingwell B.A., Dewar E.M., Reid C.M., Jennings G.L.R. (2001). Correction of carotid augmentation index for heart rate in elderly essential hypertensives. Am. J. Hypertens..

[36] Shirai K., Hiruta N., Song M., Kurosu T., Suzuki J., Tomaru T., Miyashita Y., Saiki A., Takahashi M., Suzuki K. (2011). Cardio-ankle vascular index (cavi) as a novel indicator of arterial stiffness: Theory, evidence and perspectives. J. Atheroscler. Thromb..

[37] Yamashina A., Tomiyama H., Takeda K., Tsuda H., Arai T., Hirose K., Koji Y., Hori S., Yamato Y. (2002). Validity, reproducibility, and clinical significance of noninvasive brachial-ankle pulse wave velocity measurement. Hypertens. Res..

[38] Homan T.D., Bordes S.J., Cichowski E. (2023). Physiology, Pulse Pressure.

[39] Salas-Salvadó J., Rubio M.A., Barbany M., Moreno B. (2007). Consenso seedo 2007 para la evaluación del sobrepeso y la obesidad y el establecimiento de criterios de intervención terapéutica. Med. Clínica.

[40] World Medical Association (2025). World medical association declaration of helsinki ethical principles for medical research involving human participants. JAMA.

[41] Poulton A., Pan J., Bruns L.R., Sinnott R.O., Hester R. (2019). A smartphone app to assess alcohol consumption behavior: Development, compliance, and reactivity. JMIR mHealth uHealth.

