# Supplementary material for: Alcohol Consumption Patterns and Peripheral Arterial Stiffness in Young Adults: Sex-Specific Findings from the EVA-Adic Study
Source: Nutrients. 2026 Jan 26;18(3):411. doi: 10.3390/nu18030411 (PMC12899989; doi:10.3390/nu18030411)
Supplement: Supplementary file 1 [file nutrients-18-00411-s001.zip › nutrients-4074125-supplementary.pdf]

Supplementary Materials:

Figure S1: Flowchart of the sample selection process

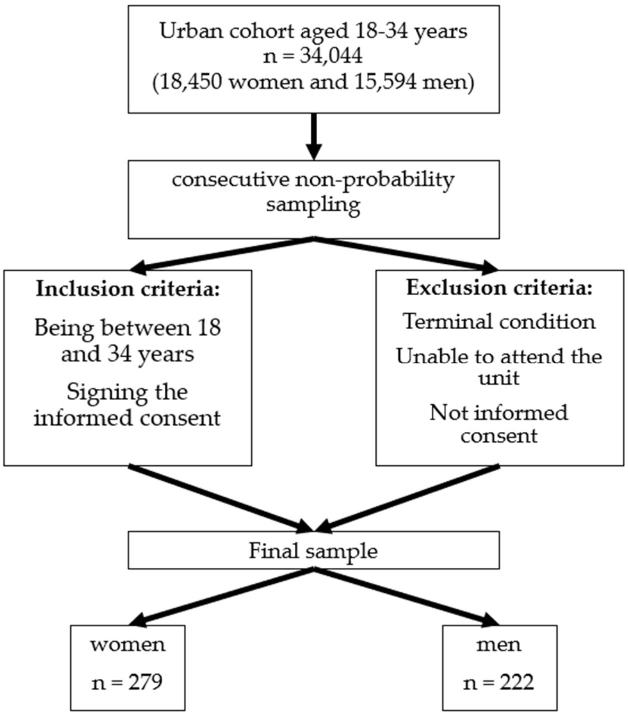

Figure **S1** displays the flowchart regarding the sample selection process for our study. The urban population aged 18 to 34 in Salamanca comprised 34,044 individuals (18,450 women and 15,594 men). From this population, a sample of 501 participants (279 women and 222 men) was obtained using non-probability consecutive sampling, applying the inclusion and exclusion criteria detailed in the Materials and Methods section.

Figure S2. Overall correlation between alcohol consumption and arterial stiffness.

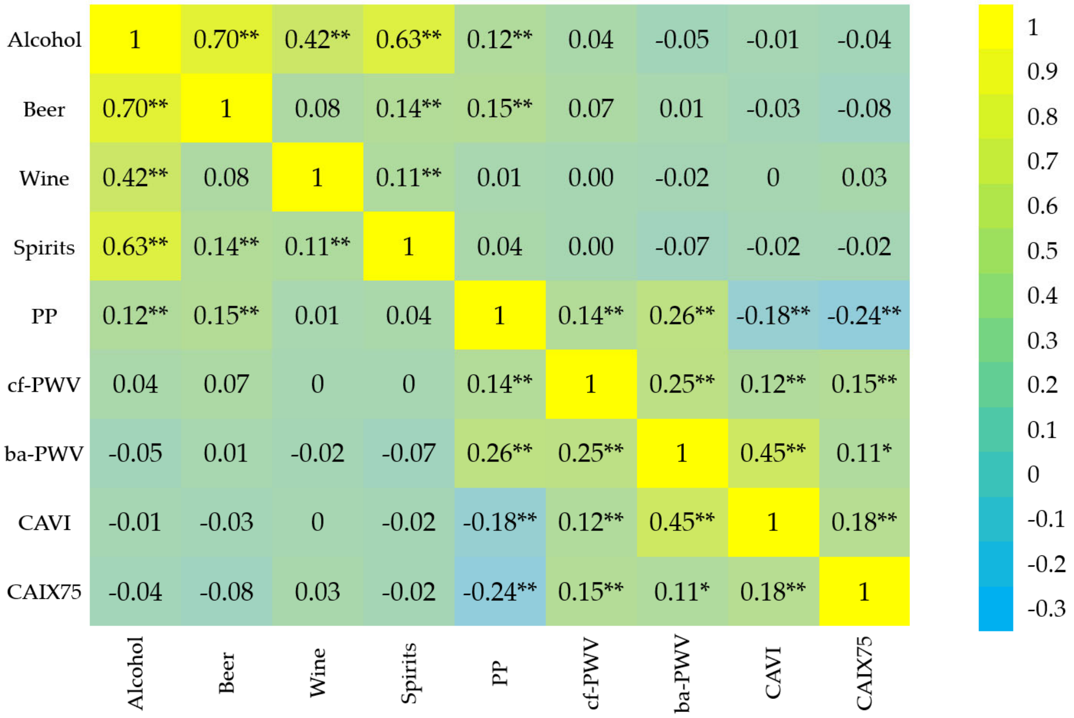

Figure S2: Overall correlations among the studied parameters. The 'Alcohol' group refers to total weekly alcohol consumption. PP: clinical pulse pressure; cf-PWV: carotid-femoral pulse wave velocity; ba-PWV: brachial-ankle pulse wave velocity; CAVI: cardio-ankle vascular index; CAIx75: central augmentation index adjusted for a heart rate of 75 bpm. Alcohol, beer, wine, and spirit consumption are expressed in grams/week. cf-PWV and ba-PWV are measured in m/s. * Correlation is significant at the 0.05 level (2-tailed). ** Correlation is significant at the 0.01 level (2-tailed).

Figure S3. Overall correlation between alcohol consumption and arterial stiffness in men.

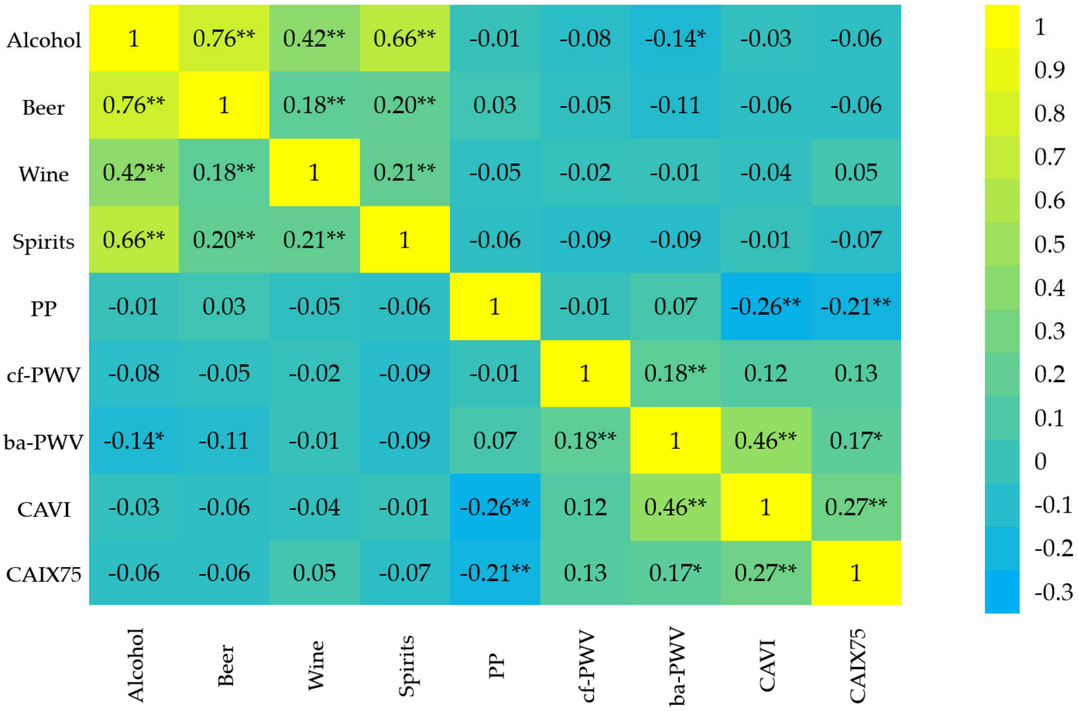

Figure S3: Overall correlations among the studied parameters in men. The 'Alcohol' group refers to total weekly alcohol consumption. PP: clinical pulse pressure; cf-PWV: carotid-femoral pulse wave velocity; ba-PWV: brachial-ankle pulse wave velocity; CAVI: cardio-ankle vascular index; CAIx75: central augmentation index adjusted for a heart rate of 75 bpm. Alcohol, beer, wine, and spirit consumption are expressed in grams/week. cf-PWV and ba-PWV are measured in m/s. * Correlation is significant at the 0.05 level (2-tailed). ** Correlation is significant at the 0.01 level (2-tailed).

Figure S4. Overall correlation between alcohol consumption and arterial stiffness in women.

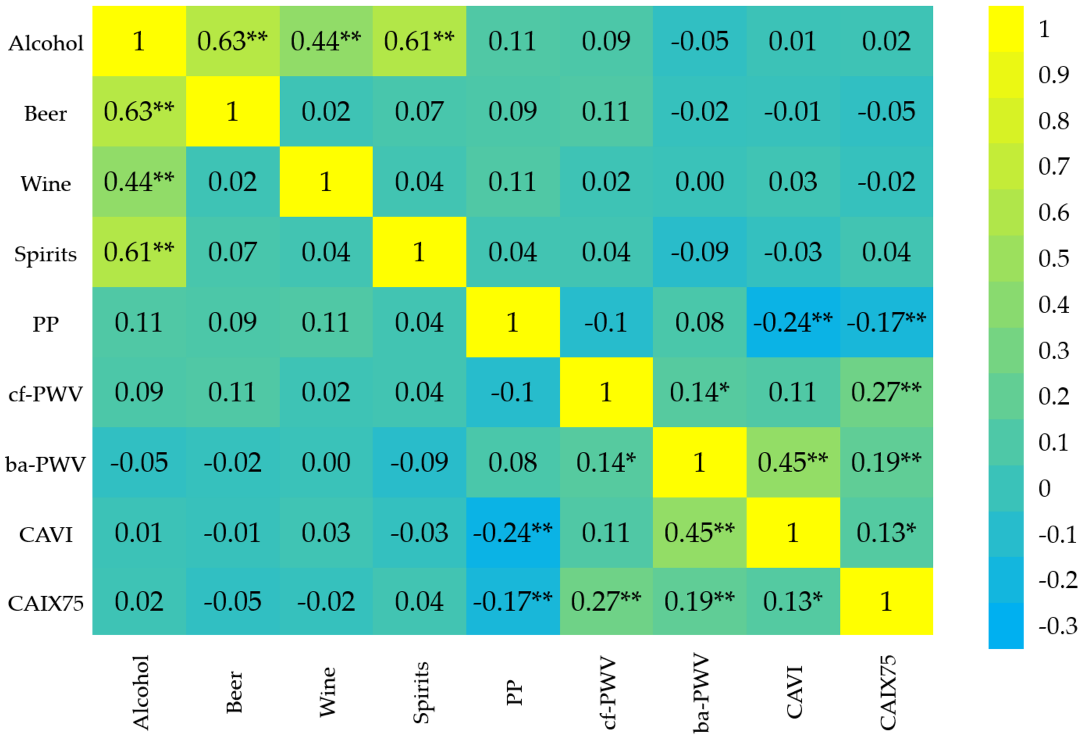

Figure S4: Overall correlations among the studied parameters in women. The 'Alcohol' group refers to total weekly alcohol consumption. PP: clinical pulse pressure; cf-PWV: carotid-femoral pulse wave velocity; ba-PWV: brachial-ankle pulse wave velocity; CAVI: cardio-ankle vascular index; CAIx75: central augmentation index adjusted for a heart rate of 75 bpm. Alcohol, beer, wine, and spirit consumption are expressed in grams/week. cf-PWV and ba-PWV are measured in m/s. * Correlation is significant at the 0.05 level (2-tailed). ** Correlation is significant at the 0.01 level (2-tailed).
